# A deep semantic matching approach for identifying relevant messages for social media analysis

**DOI:** 10.1038/s41598-023-38761-y

**Published:** 2023-07-25

**Authors:** Frederick Brown Biggers, Somya D. Mohanty, Prashanti Manda

**Affiliations:** 1 Artificial Intelligence and Natural Language Processing, United Health Group, Raleigh, NC USA; 2 Electronic Resources and Information Technology, University of North Carolina at Greensboro, Greensboro, NC USA; 3 Informatics and Analytics, University of North Carolina at Greensboro, Greensboro, NC USA

**Keywords:** Natural hazards, Socioeconomic scenarios

## Abstract

There is a growing interest in using social media content for Natural Language Processing applications. However, it is not easy to computationally identify the most relevant set of tweets related to any specific event. Challenging semantics coupled with different ways for using natural language in social media make it difficult for retrieving the most relevant set of data from any social media outlet. This paper seeks to demonstrate a way to present the changing semantics of Twitter within the context of a crisis event, specifically tweets during Hurricane Irma. These methods can be used to identify the most relevant corpus of text for analysis in relevance to a specific incident such as a hurricane. Using an implementation of the Word2Vec method of Neural Network training mechanisms to create Word Embeddings, this paper will: discuss how the relative meaning of words changes as events unfold; present a mechanism for scoring tweets based upon dynamic, relative context relatedness; and show that similarity between words is not necessarily static. We present different methods for training the vector model in Word2Vec for identification of the most relevant tweets for any search query. The impact of tuning parameters such as Word Window Size, Minimum Word Frequency, Hidden Layer Dimensionality, and Negative Sampling on model performance was explored. The window containing the local maximum for AU_ROC for each parameter serves as a guide for other studies using the methods presented here for social media data analysis.

## Introduction

Twitter is one of the preeminent microblogging platforms worldwide. With a reach of nearly 27 million Monetizeable Daily Active Users (mDAU) in the US and 126 million mDAU worldwide, Twitter users generate nearly 500 million tweets per day^1^. Twitter’s ubiquity, combined with its functionality, ease of use, and API configuration make it an frequent tool for harvesting data. Examples of this type of implementation include: pairing the metadata associated with each tweet to datasets^2^ or applying spatio-temporal metadata to isolate tweets for the purpose of analyzing regionally relevant events as they occur^3^. With the prevalence of cellphone use during emergency situations, and the above mentioned features, Twitter can be an effective asset for first responders in times of crisis.

However, determining what tweets would be considered relevant to the needs of emergency personnel presents a more challenging problem. Challenging semantics coupled with different ways for using natural language in social media make it difficult for retrieving the most relevant set of data from any social media outlet. Tweets can contain any manner of content, be it observations of weather related phenomena, commentary on sports events, or social discussion. Isolating relevant tweets requires analysis of a multitude of characteristics such as location and time based metadata, but also the content of the tweet itself. With events occurring in varying locations, each with their own regional parlance, metalinguistics, and iconography, while addressing the meaning(s) of text changing relative to the circumstances at hand, a dynamic interpretation of linguistics is necessary.

The aim of this study is to test methods and associated parameters for optimizing context analysis for event related semiotics within tweets generated during emergency events. We analyzed and compared methods for Word Embeddings using vectorization in tweets. A series of Neural Networks were trained via Word2Vec to convert words in tweets into numerical representations of meaningful context relationships. These contexts were then applied to find tweets which were connected to designated search terms. The resulting processes were used to identify a more comprehensive set of related tweets beyond those indicated by the presence of the initial search term(s). Findings from this work can be applicable for emergency response personnel who seek to retrieve geolocated tweets associated with disasters, without using a predetermined set of search criteria.

This study used tweets generated during Hurricane Irma to demonstrate our methods. Hurricane Irma made landfall on the Florida coast on September 10, 2017 as a Category 4 storm^4^. Rain and wind resulted in a storm maximum of “...21.66 inches of rain... measured between 9 and 12 September...”^4^ and “...produc[ing] 25 confirmed tornadoes: 21 in Florida and 4 in South Carolina.”^4^. Hurricane Irma, as of 2017, was the fifth most costly Tropical Cyclone to hit the United States, with an estimated cost of damage nearly $50 billion^5^.

The contributions of this work include methods to determine comparative relatedness between a single word and a micro-blog post (e.g. Twitter, etc.) within a temporal context, i.e. can the meaning of a tweet be derived when the time of composition can affect the interpretation of what is said. To do this requires a two-fold approach. The first is predicated upon the premise that large comprehensive corpora must rely upon a probabilistic determination of meaning for homonyms. That is to say, absent extensive context, a word with two disparate meanings may be interpreted incorrectly if one meaning occurs more frequently within a corpus than the other. This model addresses this by training on smaller, more concise corpora. Secondly, as word embeddings rely upon a vector to describe meaning, this paper attempts to determine the best linear operations for comparisons of a single word embedding to multiple word n-grams within the same vector space. The result is a real number score of relatedness, minmax scaled to 0–100.

## Background

### Word embeddings

Word embedding is the generic term for assigning numeric values to words, with the mathematical operations between those numeric values implying some semantic or syntactic relevance^6^. These numeric values are assigned based on a computer generated algebraic representation of observed contextual relationships. Such representations are critical in designating syntactic intent in a manner such that it is capable of being interpreted by a computer. To provide this function within such a model, word embeddings must be created based upon an algorithmic approximation of natural language. Without such a framework, words would lack the necessary connections to each other.

Numerical values must therefore be established based upon a uniformly consistent translation encapsulating context and meaning between words. This process is defined as isolating commonalities between words, determining a dimensional model capable of representing relationships between these words, and assigning numeric values to words based upon their individual spatial locations. Each word then has a corresponding vector within this dimensionality. This vectorization of words thus embeds meaning into these numerical representations.

### Training corpus

Training a computer to determine word meanings requires a sufficient and relevant body of text. This body is known as a corpus. It is important that a corpus be similar in purpose to the text that is intended to be analyzed. To clarify, an algorithm trained on text retrieved from business emails may not be adequately trained to determine ingredients in cookbooks. As illustrated in Yang et al., analysis of Twitter content by a neural network trained on “aligned” content performs better than a neural network trained on a Wikipedia dump^7^. Likewise, the meaning of an individual word is governed by its context; inconsistency across contexts can introduce an element of ambiguity, thus reducing the effectiveness of machine learning.

In the case of Twitter, the process of training via a corpus must be done with allowances to compensate for linguistic variations in grammar and syntax, as well as restrictions due to character limits. In addition to these variables, topics within Twitter can trend and the meaning of words can change based upon dominant topics. Tweets generated during a natural disaster, such as a hurricane, can change the context of concepts and words (e.g.: the difference between literal: *there is a flood on my street* and metaphorical: *a flood of tears*). As word relationships can often be derived from the relative placement of words, the context in which these words appear will add another potential avenue of complexity to the vectorization process.

Searching for tweets associated with a named occurrence, such as a natural disaster, can yield artificially limited results even when the name is used as part of the search criteria. For Twitter to provide data to emergency responders during a natural disaster, a system must be employed to help isolate tweets that are relevant to that event. Training such a system for natural disaster context recognition requires a body of temporally relevant data. Once this training is complete, a metric must be implemented by which the relatedness of terms or text can be evaluated.

If contextual information contained in tweets is to be relevant to emergency responders, two primary factors must be addressed. The first factor is that the semantic accuracy of any given system of analysis is relative to the topics trending at that point in time. The overall meaning of a given tweet is dependent on how the words it contains are used under immediate circumstances. Changes in topics or contexts influences the interpretation of individual words^8^. Static training of machine learning systems on enormous corpora is effective for probabilistic interpretation of consistent meaning across a uniform body, but lacks the nuance necessary for interpreting polysemy as it changes from moment to moment.

The second factor is matter of available resources. It is important that the analysis functionality of this system be efficient at a level of computational infrastructure investment attainable in situations where funds and capability are limited on short notice^9^. Again, while corpora of millions or billions of lines of text are necessary to train more universal text recognition machine learning models, their efficiency can often be measured in hours or days^10^. The typical response in cases of emergency must be significantly shorter.

## Related work

### Social media as a crisis resource

Social media has been shown to be an effective means of addressing crisis events^11,12^. The study and responsive analyses of social media and its applicability to crisis events has been termed crisis informatics^13,14^. Crisis informatics can encompass natural disasters, such as floods^3^, hurricanes, and wildfires^11^, or can be applied to social and medical crises such as opioid addiction^15^ and the spread of disease^12,16^.

In the study of crisis informatics, social media can function as part of the toolset used in crisis preparation and emergency preparedness^17^; and for response and communication during the event^18–20^. Poblet et al. describe the roles of social media separated across distinct data types as a crowdsourced, multi-tiered tool^18^. Social media can be used as a source of data, because it can function as the product of the “crowd as a sensor”^18^ by providing location data or other metadata that can be correlated with known datasets “...especially in the mitigation and preparedness phases [of disaster management]”^18^. Of particular interest is the “crowd as a reporter”^18^, wherein social media users report “first-hand information on events as they are unfolding” to a specific social media platform^18^.

Reporting data to a social media platform is the first component of the crowd as a sensor. Reuter et al. categorizes interaction aspects of communication within crisis informatics into four categories: Authorities-to-Citizens (A2C), Authorities-to-Authorities (A2A), Citizens-to-Citizens (C2C), and Citizens-to-Authorities (C2A)^14^. In the C2C quadrant, communications are categorized as “Self-Help Communities” where private citizens are sharing crisis-related information relevant to their locality; this data is intended for other regionally coincident private citizens and is not specifically broadcast to, or for, emergency responders^14^. Finding and assessing user-generated social media content intended either for other citizens or authorities in times of crisis, without necessarily distinguishing between the two, is essential to this study.

These papers focus largely on the use of social media as “sensors”, where individuals on the ground during crisis events can be leveraged to provide information. These individuals are not necessarily official responders, yet their information can be reliable when properly processed. While this paper agrees with the assessments of this work, it seeks to expand upon their research and provide a possible method for parsing social media information in a rapidly changing context.

### Natural language processing and text mining

Social media content, like that contained in Twitter, exhibits many of the pitfalls of processing natural language and presents unique challenges depending on objective.

One way to mine data largely comprised of natural language is to correlate the unstructured content with more structured datasets via unique identifiers and metadata. Longley and Adnan have leveraged both the structured and unstructured data in Twitter to produce effective demographic analyses in London^2^. In their study “...represent[ing] a small and self-selecting sample of all Twitter users in London...”, their methods were used to correlate geo-temporal metadata with other datasets, and employ natural language processing techniques to determine ethnicity, age, residence, and commuting routes, among other demographic data^2^.

In cases where consistent semantic interpretation over a large number of documents is important, methods have been employed to increase the immutability of the vocabulary. In Pedersen et al. one such mechanism is to reduce the vocabulary, while minimizing the reduction’s impact on meaning^21^. This has been accomplished by swapping words within an acceptable range based upon semantic similarity^21^. Priority is placed upon enforcing semantics in an absolute sense, where the meaning (or meanings) of a word remain relatively static within the context of the document, e.g. where bi-grams like *heart attack* should be correlated with *myocardial infarction* or *coronary thrombosis*^21^. Analysis on semantics, therefore, can be compared across the entire corpus despite similar concepts being represented by analogous phrases.

In these works, the authors aim to analyze and correlate social media data, specifically Twitter, to accommodate multiple uses. Different techniques are employed to widen the capabilities of analysis, but depend on significantly larger datasets. The aim of this paper is to increase the flexibility of the systems employed by deliberately reducing the amount of input data. The assertion here is that a reduction in data input increases the likelihood of the algorithm being able to interpret relevant meaning specific to the events as they occur.

### Using Word2Vec to create embeddings for Twitter data

Many studies have approached analyzing the semantic content of Twitter data by using Word2Vec as a mechanism for creating word embeddings. In Yang et al. Word2Vec was employed with various tests of hyperparameter values for analysis of tweets related to an election^7^. This study compared the effectiveness of training Word2Vec neural networks on Spanish Wikipedia with those trained on Twitter data sets. Their training data was labeled as “election related” or “non election related” and focused on tweets that occurred during a parliamentary election in Venezuela in 2015. Their objective was to attempt to predict whether a tweet could be identified as election related based upon the vector representations of words contained in the tweet. The study found that training on an aligned data set (using Twitter data instead of a more generalized corpus, such as content from Wikipedia) and proper configuration of Word2Vec parameters (specifically increased word/context window and dimensionality sizes) proved effective at creating representations of the tweets themselves^7^.

In Benton et al.^22^, Word2Vec was one of the components used to create vector representations based upon the text of Twitter users. In their study, the intention was to create embeddings to illustrate relationships for users, rather than words, and then use these embeddings for predictive tasks. To do this, each user “representation” is a set of embeddings aggregated from “...several different types of data (views)...the text of messages they post, neighbors in their local network, articles they link to, images they upload, etc.”^22^. The *views* in this context are collated and grouped based upon the testing criteria. For example, to predict user created content, a view of tweets created by a particular user would be isolated, and the neural network trained on the user’s tweets as a single document.

In this section, previous authors have demonstrated that Word2Vec is capable of analyzing the text of tweets. In one case, this is determined by using a narrowly defined set of related tweets to classify a tweet as election related. While the objective here is similar, the approach for this paper is to provide a mechanism for broader search criteria, not necessarily restricted to a single event. By training on data contemporaneous with potentially relevant search criteria, the algorithm seeks wider capability and flexibility, both in its interpretation of meaning and relevance.

### Recent applications of neural networks for social data analysis

While the data used in this study does not require privacy accommodations, other kinds of data might necessitate privacy-aware methods. For example, user location is collected on social networks through cell phones, wearable devices, etc. Qi et al.^23^ presented a point-of-interest category recommendation model that is privacy-aware. LSTM-based neural architectures are used for recommendations and users are classified into similar groups via hashing to protect user privacy. Other improved methods used graph convolution networks that can learn the dynamic relationships between users and points of interest^24^. LSTM-based models have shown promise in another application for analysis of sensor data. Prediction models to understand the climate of a greenhouse for robust crop production have shown utility for farmers^25^.

## Methods

### Corpus creation

Tweets for the time period 2017-09-10 00:00 GMT through 2017-09-11 00:00 GMT, inclusive were retrieved. This dataset was obtained from previous projects including a seed grant project for the Giant Steps program at UNC Greensboro. Of the 784,322 tweets used for the comprehensive project, this paper focuses on the 19,088 tweets composed during the 24 h period starting at 2017-09-10 00:00:00 UTC and had an encoded language of English. For reference, Hurricane Irma made landfall in Cudjoe Key, Florida around 1300 UTC, roughly the midpoint of the time span used to isolate relevant tweets.

These tweets were human-coded for relatedness to Hurricane Irma. For purposes of identifying relatedness, a tweet whose context was interpreted by a human reader as being associated with Hurricane Irma was given a boolean True value. These tweets were then isolated to exclude non-English content. To ensure independence between human-coded data and the training mechanism, the value for human-coding was not introduced into the neural network as a feature during training. This attribute was only used when evaluating the effectiveness of a particular scoring formula, and to assess the impact of variation on a parameter. We note that coding the tweets using only one human coder introduces a certain amount of bias into the coding.

### Tokenization and cleaning

Text in the corpus was first processed using regular expressions and tweet tokenization functions. One of the libraries leveraged for this process is NLTK, the Natural Language Toolkit. The NLTK reduce_lengthening under nltk.tokenize.casual will reduce concurrent repeated characters to three incidents. For example, ‘OOOOOMMMMGGGGGGG’ would be reduced to ‘OOOMMMGGG’. It is assumed that homographs separated only by character quantity could be reduced to the same word. This operation decreases the overall vocabulary size, with minimal impact on individual token meaning.

Further token removal for stopwords was performed by removing entries in the NLTK English stopwords library. The Frequent Word Subsampling function in the Word2Vec specification was used to remove frequent terms from corpora based upon frequency, as opposed to a static list of words observed to add no additional syntactic import.

The terms were cleaned using regular expressions, and a custom cleaning function was defined to remove the following from all tweets:

1. Uppercase letters
2. URLs beginning with http:// or https://
3. @mentions, including those with a leading ’-’ or ’.’
4. Punctuation, but not hashtags (#)
5. Non-hashtag # (e.g. bounded on left by word character, single-character instance, etc.)
6. Word-bounded numbers
7. encoded HTML

While there are incidents where character case might denote semantic difference, such as march (to travel in regular pattern) or March (the third month), patterns of case vary widely through tweets. As strings containing URLs impart no semantic value to text, any appended URLs were stripped from text. Once cleaned as above, the remaining word tokens were processed through a stemmer function. The purpose of the stemmer is to further eliminate redundancy in the vocabulary, by treating words with the same stems as semantically equivalent. The words *heavy*, *heavier*, and *heaviest* would be reduced to *heavi*.

#### Word2Vec and parameters

The Word2Vec vectorization method has been shown to be an effective way to derive meaning from a large corpus, and then use that meaning to show relationships between words^10,26,27^.

To begin this process, the vocabulary of the corpus is defined and its size determined. Details for dimensionality reduction and training can be found in Ref.^27^.

Backpropagation occurs via stochastic gradient descent, and the process begins again with the next word within the context window. Once all context terms are processed within the word window for the center word, the process begins again with the next center word and its context words.

### Cosine similarity

Once the vectors have been constructed in a manner where spatial relationships imply syntactic relevance or similarity, mathematical comparisons of these vectors can be used to interpolate meaning. In the vector dimensional space of word embeddings, vectors of words with similar context or meaning will tend to congregate. One way to quantify vectors’ spatial proximity can be done by comparing their internal angles.

The cosine trigonometric function has the property where two coincident vectors will have a cosine of 1, as their internal angle has a measure of zero. As two vectors diverge, their internal angle increases. An internal angle measure of 90$^\circ$ has a cosine of zero. Between 0$^\circ$ and 90$^\circ$, the cosine of the angle has a real, positive value between one and zero, respectively.

As the angle continues to increase above 90$^\circ$, and up to 180$^\circ$, there is a commensurate relationship with the cosine of this angle as well. The cosine of 180$^\circ$ has a value of negative one, and the cosine of the angles between 90$^\circ$ and 180$^\circ$ have a range of real, negative values between zero and negative one.

Envisioning each term within the context of a corpus as having a vector, and that vector’s spatial position related to the term’s context or meaning allows the relatedness of two vectors to be interpreted as inversely proportional to the degree of the internal angle formed by the two vectors.

Thus, the phrase *cosine similarity* is used as a real number representing how close two terms are within the context vector space. Two similar or related terms will have a cosine similarity as a real value close to one, where two lesser-related terms will have a lower cosine value, to a minimum at negative one.

### Word2Vec parameters

#### Vector training methods

In the Word2Vec module, there are two different methods of training the vector model, and they are nearly opposites of each other. The first, Continuous Bag-of-Words (CBOW) trains the neural network by using the context words as the input and the expected target word as the output. The intended use here is to predict a single word based upon an input of one or more context words.

The other method for training the neural network is the Skip-Gram model. In this model, the center word is the single input; the context words are the output. This model aims to predict context words based on a single word.

The neighboring words are also scored by their relative location to the center word, and weighted with a proportional function to emphasize a context word when it is closer to the center word. In this way, a context word that is directly adjacent to the center word carries more weight for context than a word that is a few positions away^26^.”

Both methods are built upon maximizing the probabilistic pairing of the correct word, *w*, with the correct context *c*. The difference comes from the conditional event notation: $P(w \vert c)$ indicates the CBOW relationship, while $P(c \vert w)$ indicates Skip-Gram, for any given word-context pair (*w*, *c*).

#### Minimum word frequency

Word frequency can play an important role in analysis of large bodies of text. Setting a floor on the occurrences of a word below which it is ignored can prevent a word from being included in the vocabulary entirely. This can be important if a corpus contains jargon or slang that is not necessarily endemic to the work(s) in question. It is possible, however, that too aggressive of a floor on occurrence frequency could diminish some of the nuanced meaning desired by this study. Furthermore, wholly unique tweets could be eliminated from consideration entirely.

#### Word window

The word window argument sets the maximum distance on either side of a center word where neighboring words are considered for context. For example, a word window of 3 would look both three words ahead and behind the center word to include any words found in the context part of the neural network construction. Though words outside of this window are considered to be part of the same document, words within the same document will share context words where the word windows overlap. For CBOW, these words are the input values for the neural network, and for Skip-Gram, these words are the output values.

#### Word vector/hidden layer dimensionality

The construction of the neural network is based upon inputs and outputs, but the internal weights are used as a representation for each of the word embeddings^27,28^. For the purpose of this project, the dimensionality of the word embedding vectors and the hidden layer of the neural network are equivalent, and the terminology will be used interchangeably.

#### Negative sampling

If all words in a vocabulary *V* are combined such that $$\left( {\begin{array}{c}V\\ 2\end{array}}\right)$$ represents all possible word-context pairs, far more pairs exist than true word-context relationships within the training corpus.

If the neural network is only trained on all valid word-context pairs pairs in *N*, then any single pair has tremendous significance. The parameter for the negative sampling function, *k*, indicates a choice of *k* negative values that limits the impact of any single pair^29,30^.

By default, the Gensim implementation of Word2Vec for Python uses a negative sampling value of 5, where the recommended range is 5–20^28,30,31^.

As the objective for training involves numerous rows on both the input and output layers, the update equations must be similarly adjusted^27^.

#### Scalar comparison formulas

After training, the Word2Vec neural network produces vectors for terms but not tweets. For the results of this analysis to be compatible with the other scoring mechanisms, a single scalar value would need to be determined for each tweet. The following formulae were used to derive a scalar score for the tweet from an amalgamation of the component term vectors. In the initial testing, each formula was executed in tandem, and the equations would be used to compare the effect of variation in the parameters. For purposes of consistency, and to distinguish from previous terminology, new symbols will be used for the components necessary for these comparisons. The symbol $\alpha$ designates the initial search or *seed* term, the basis of all comparisons for these formulas. The symbol $\tau$ will refer to a token contained within a processed tweet, where $\tau _i$ indicates one of many such tokens in any given tweet.

*Cosine Similarity From Cosine Distance of One Dimensional Arrays (CSTVS)* The SciPy spatial.distance library has a built-in function for cosine distance between two 1D arrays, interpreted as vectors.

The cosine distance between the matrix-as-vector and the word vector for the seed term *irma* is calculated. Cosine distance can be further converted to cosine similarity by subtracting from one. This formula was selected to leverage the efficiency of optimized pre-generated code over other possible functions. If the performance of this scoring mechanism proved to be nearly equivalent to others of the formulas, then it could be evaluated on the basis of resource and time consumption.

### Dot product of search term vector and tweet vector sum (DP)

Cosine similarity is proportional to the dot product of two vectors. It has been observed within the vector constructs for Word2Vec that vector operations, such as addition and subtraction, yield meaning^10,26^. This was used as the predicate for interpreting the meaning of a tweet as the sum of its component word vectors. Summation of all of the token vectors, $\tau _i$, within a tweet returned a vector itself in the same dimensionality as, and therefore could be compared to, the vector for the seed term *irma*, $\alpha$, via the cosine similarity of the two. The dot product operation gives a scalar value for the tweet comprised of related word vectors.

### Mean cosine similarity of tweet terms in vector vocabulary (MCS)

1 $$\begin{aligned} \frac{1}{n}\sum \limits _{i=1}^n \tau _{i}. \end{aligned}$$

For this process, after tokenization and cleaning, each remaining token, $\tau _i$, in each tweet was scored based upon its cosine similarity to the seed term *irma*. If a term was not present in the vocabulary, due to minimum word count or other restricting criteria, the term was given a zero, which evaluates to a neutral context relation due to cosine similarity. The mean of all cosine similarity values for tokens $\tau$ within the tweet, including zeroes, was calculated, and this value was designated as the score for the tweet.

### Sum of cosine similarity of tokens over square root of token count (SCSSC)

2 $$\begin{aligned} \frac{1}{\sqrt{n}}\sum \limits _{i=1}^n \tau _{i}. \end{aligned}$$

The sum of cosine similarity of tokens scores a tweet based upon a summation of the tweet’s component token vectors. However, the scalar value calculated using mean cosine similarity could disproportionately favor shorter tweets, as each token would contribute a greater proportion of the score. In an attempt to minimize the impact of word count in any given tweet, the mean operation was replaced by dividing by the *square root* of the word count.

## Results

A total of 19,088 tweets were identified in the corpus. The tweets were cleaned, processed, and tokenized. The following Table 1 shows the twenty most frequent tokens and their counts prior to any transformations.

**Table 1:** Pre-transformation count of tokens in tweets.

Token	Token count
The	4900
I	4133
To	3853
@	3337
a	3020
In	2998
And	2843
Of	2796
Is	2619
For	1977
My	1943
s	1772
You	1647
Florida	1592
This	1572
On	1491
t	1357
From	1236
It	1202
At	1129

The first transformation performed was the reduce_lengthening functionality. This function reduced the total number of tokens by $.31\%$. Any superfluous tokens decrease the effectiveness of training; two (or more) words whose existence otherwise would be treated as identical, but whose spelling is only separated by the quantity of a character, and therefore completely different, dilutes the likelihood of the neural network recognizing their syntactic equivalence.

Stopword removal, frequent word subsampling, and further cleaning using a custom function detailed in “Tokenization and cleaning” were performed. Once these operations were performed, the number of tokens was further reduced by 66.9% leaving 14,439 tokens. The list in Table: 2 shows the top twenty words ordered by count after the combined transformations. When compared with the initial list in Table: 1 it is immediately apparent that case-sensitivity is significant in minimizing vocabulary. In the first table, *Florida* occurs 1592 times. After cleaning, *florida* appears 1809 times and is the most frequently used word. Note: the incident of the word *hurrican* could likely be attributed to misspelling, but also to the effect of the stemmer function (i.e. truncating both *hurricane* and *hurricanes* to their root).

**Table 2:** Post-transformation count of tokens in tweets.

Token	Token count
Florida	1809
#Hurricaneirma	1623
fl	1587
Irma	1374
Hurrican	1360
#Irma	1193
Wind	946
Get	936
Report	886
Go	830
Storm	775
Power	715
Miami	705
Rain	682
mph	661
Like	657
Beach	656
Gust	655
Safe	633
Aso	544

**Figure 1:**
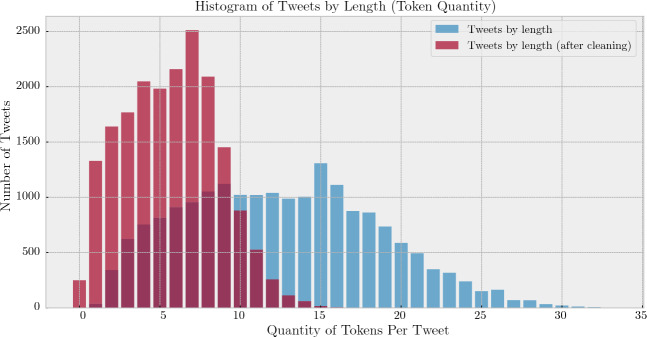
Histogram of tweets by length (token quantity).

The graph in Fig. 1 shows the quantity of tweets by number of tokens before and after processing.

Tweets in the corpus had a maximum length of 33 tokens, separated by whitespace characters, prior to cleaning and tokenization. The performance of these operations reduced the maximum number of tokens in a tweet to 20. 83.7%, contained 10 or fewer tokens.

### Selection of scalar formulas

The initial sets of tests compared the AU-ROC of each scalar formula as applied to tweets relative to the search term: *irma*. Each iteration of testing involved training the neural network with default values for each parameter, isolating one parameter and determining a window which contained a local maximum for AU-ROC.

#### Tuning parameters

##### Word window size (WWS)

 The initial test for the Word Window Size parameter variability set a ceiling at 10 tokens on either side of the center word. The other parameters were set at constants: minimum word count 1, word vector dimensionality 100, negative sampling 5, and using the Skip-Gram model. The maximum token count for a tweet within this data set was 20. A word window value of 10 as the upper bound for the testing range ensured that all center words were provided at least half of the encompassing tweet as context. This also ensured that any given word potentially had the entire tweet as context for 83.7% of tweets. See Fig. 1 for the distribution of tweets by length.

**Table 3:** AU-ROC of word window size values 1–10.

Scalar comparison	Word				Window	Size				
	1	2	3	4	5	6	7	8	9	10
CSTVS	0.71	0.71	0.72	0.74	0.73	0.74	0.75	0.75	0.75	0.75
DP	0.81	0.81	0.82	0.82	0.82	0.82	0.82	**0**.**82**	0.82	0.82
MCS	0.51	0.55	0.59	0.62	0.62	0.63	0.65	0.66	0.65	0.66
SCSSC	0.72	0.72	0.74	0.76	0.76	0.77	0.78	0.78	0.78	0.79

For word window values 1 through 10 in Table 3, the four scalar comparison formulas have a maximum observed AU-ROC at window size 8 for the Dot Product formula. While the difference in scores was negligible, it did indicate a trend towards a local maximum, therefore further tests were not performed.

##### Minimum word frequency (MWF)

Testing Minimum Word Frequency presented a different problem than most of the other parameter tests. By setting a threshold on frequency, it would be possible for a tweet to be comprised entirely of words that would not exist in the vocabulary of the vector sets. With the scalar comparison formulas dependent on the cosine similarity of a term and the search term, if a vector did not exist, it is possible for some of the tweets to end up with component elements in the denominator equal to zero. This required additional error handling in the code representing the scoring formulas.

Variation in Minimum Word Frequency also affected the maximums for each scalar comparison formula differently. With each of the other parameters, the maximum AU-ROC score consistently correlated with the same value for all scalar comparison formulas (e.g. the optimal value for Word Window Size, 8, corresponded to a maximum AU-ROC for all four formulas, see Table 4). With Minimum Word Frequency, the optimal value for three of the four formulas was 8. However, for the Dot Product formula, the optimum value for Minimum Word Frequency was 3.

**Table 4:** AU-ROC of minimum word frequency values 0 to 9.

Scalar comparison	Word				Frequency					
	0	1	2	3	4	5	6	7	8	9
CSTVS	0.74	0.73	0.74	0.74	0.73	0.73	0.74	0.74	0.75	0.73
DP	0.82	0.82	0.83	**0**.**83**	0.82	0.82	0.83	0.83	0.83	0.82
MCS	0.63	0.62	0.63	0.63	0.62	0.63	0.64	0.64	0.66	0.63
SCSSC	0.77	0.76	0.78	0.79	0.79	0.79	0.80	0.80	0.81	0.79

##### Hidden layer dimensionality (HLD)

 As with the previous tests, the Dot Product formula indicated the best performance for scoring a tweet. Changes in vector dimensionality yielded minimal performance changes, as indicated in Table 5. All formulas performed best with a dimensionality of 150, though the change from the default 100, showed little appreciable difference in the results.

**Table 5:** AU-ROC of hidden layer dimensionality values 50–450.

Scalar comparison	Hidden				Layer	Dimensionality		
	50	100	150	200	250	300	350	400
CSTVS	0.74	0.75	0.75	0.75	0.75	0.75	0.79	0.75
DP	0.82	0.82	**0**.**823**	0.82	0.82	0.82	0.82	0.82
MCS	0.65	0.66	0.68	0.65	0.65	0.65	0.65	0.65
SCSSC	0.78	0.78	0.78	0.78	0.78	0.78	0.78	0.78

##### Negative sampling (NS)

The initial test of the negative sampling set out to compare the effectiveness of increased numbers of negatively sampled terms. The default value of 5 seemed to have minimal impact on the AU-ROC score. However, this test showed one of the more dramatic outliers for AU-ROC score over all tests of parameters. Changes from one value to the next for all parameter tests were measurable, but the variation rarely exceeded 0.02 in the subsequent calculation of AU-ROC (see Table 6). The difference between 0 and 1 for the negative sampling value showed a substantial increase from 0.560 to 0.854 for the Dot Product Formula. Similar increases were noted for the other scalar comparison formulas. The 0.854 for the Dot Product formula below also represents the highest AU-ROC score for all parameter tests. The remaining AU-ROC values for 2 through 9 negatively sampled words were also greater than the corresponding value for 0. This indicated that including a minimal number of negative context words in the training has an overall positive effect on the accuracy of the neural network.

**Table 6:** AU-ROC of negative sampling values 0 to 9.

Scalar comparison	Negative				Sampling	Value			
	0	1	2	3	4	5	6	7	8
CSTVS	0.56	0.77	0.75	0.75	0.75	0.75	0.75	0.76	0.75
DP	0.56	**0**.**85**	0.83	0.83	0.82	0.82	0.82	0.82	0.82
MCS	0.56	0.72	0.68	0.66	0.65	0.66	0.65	0.66	0.64
SCSSC	0.56	0.82	0.79	0.78	0.78	0.79	0.78	0.79	0.78

### Optimized parameters and grid search

Once ranges containing a local maximum for individual parameters on the AU-ROC score were determined, these ranges were used as the testing values of a Grid Search, with one alteration. With minimal initial impact seen by variability in Hidden Layer Dimensionality, only vectors of 100D and 150D were tested. Table 7 shows the top performing permutations of parameters.

**Table 7:** Grid search parameter results.

AU-ROC	HLD	MWF	WWS	NS	EP	SF
0.887560	150	5	1	1	25	DP
0.886191	100	5	1	1	25	DP
0.881556	150	3	1	1	25	DP
0.879418	150	7	1	1	25	DP
0.879235	150	6	1	1	25	DP
0.878688	150	8	1	1	25	DP
0.878547	100	6	1	1	25	DP
0.878196	100	3	1	1	25	DP
0.877670	100	7	1	1	25	DP
0.877067	150	9	1	1	25	DP

As expected, the Dot Product (DP) scalar formula performed the best overall. The Negative Sampling (NS) parameter value also reflected the observations in initial testing; a value of 1 was clearly optimal for this training. Another expected outcome was the apparent negligible impact in using 100D versus 150D for Hidden Layer Dimensionality (HLD).

The remainder of the parameters appeared to deviate somewhat from the values seen as local maximums in the initial testing. Minimum Word Frequency (MWF) and Word Window Size (WWS) were apparently affected by the simultaneous adjustment of other parameters, as well as being somewhat more influenced by the number of training epochs (EP).

**Figure 2:**
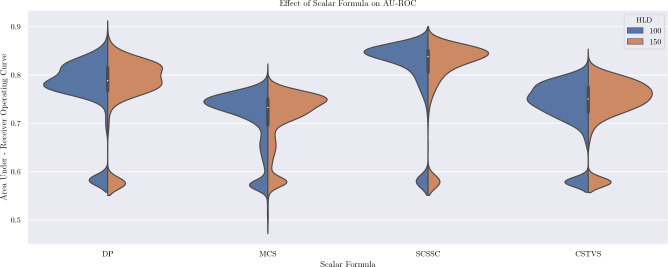
Effect of scalar comparison formula on AU-ROC.

The violin plot below (Fig. 2) shows the distributions of AU-ROC scores for each of the four scalar formulas. The two halves of each distribution correspond to the two values tested for Hidden Layer Dimensionality.

The Dot Product (DP) scalar formula shows a higher overall maximum, although with slightly greater variance, when compared to the Sum of Cosine Similarity of Tokens over Square Root of Token Count (SCSSC) (2).

#### AU-ROC of scalar comparison formulas

**Figure 3:**
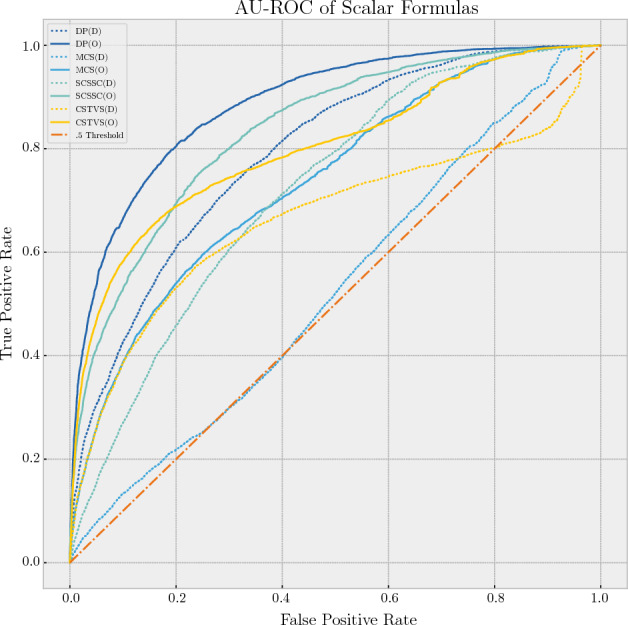
AU-ROC of scalar comparison formulas.

Using the neural network trained with optimal parameters, the tweets were again scored and their AU-ROC curves created. Figure 3 shows the scalar comparison formulas both with optimal parameters (indicated by **(O)** and solid lines) and default parameters (indicated by **(D)** and dotted lines), color-matched, with a reference line for the 0.5 AU-ROC threshold. As was indicated in previous tests, the Dot Product (DP) formula proved to be the most effective and consistent method for scoring a tweet. The Mean Cosine Similarity score seemed the least effective, but somewhat more consistent than the Cosine Similarity of Tweet Vector Sum (CSTVS). It is worth noting that dividing by the square root of the tweet length (SCSSC) proved to be a significant improvement over the simple mean.

### Dynamic relatedness

#### Word lists per hour

Once the process for training the neural networks was established with optimal parameters, it could be applied to further subdivided time deltas. In the tables below, rather than train on a full 24 hour period, each segment represents the training on tweets over a one hour period. Each list represents the top twenty most related words to the search term ‘irma’ for that hour (EST). Each word is paired with its vector’s cosine similarity to the vector for ‘irma’. These scores are the raw cosine similarity, and have not been min-maxed for their relative time delta.

Tables 8, 9, 10, and 11 show related word lists where each column represents 1 h within in the 24-h period starting at 00:00 GMT on September 10, 2017. For each hour, the Word2Vec neural network is trained on only the tweets that occur during that period, using the optimal parameter configuration determined by the grid search “Optimized parameters and grid search” above. The list of words represent the top twenty most similar by cosine similarity in descending order as compared to the search term: “irma”.

Some of the interesting observations come from interpreting the possible context and reasoning for why certain terms are positioned in lists at particular times. For example, the word *shelter* appears in various locations throughout the lists. Perhaps more interestingly, it is the top word of the hourly list at the time of Hurricane Irma’s landfall, and the top word for the subsequent three hours. And *landfal*, the stem of landfall and landfalls, only appears once: during the landfall hour. The word *tomorrow* appears four times in the five hours, 00:00–04:00. Since local time is UTC-4, these hours correspond with 8:00 p.m.–midnight on the day previous to landfall. *Tomorrow* does not appear on the lists for related words on the day of landfall.

The word *ese* presents another interesting linguistic observation. While this word has colloquial meaning in Spanish, its appearance in these lists is indicative of another meaning. Searching the graph of word communities (see: 5.3), *ese* is found in a group of weather terms. By isolating the training to English only tweets, the meaning appears to have tended toward ESE, an abbreviation for *East by Southeast*. In this context, the probability of this particular interlingual homograph was higher when considering the direction from which the hurricane approached. Furthermore, when looking at the hourly lists of words, it appeared in the top four words in each of the four hour lists prior to landfall; only once in the lists prior to that; and never in the hours afterward.

Another word that has a fascinating set of positions on this list is the word *safe*. It appears only once in the twelve hourly lists prior to landfall, at the bottom of the 08:00 a.m. UTC list. However, it appears seven times in the eleven hourly lists after landfall.

**Table 8:** Related Words 00:00 UTC–05:00 UTC.

00:00	01:00	02:00	03:00	04:00	05:00
Tampa	Whole	Time	Shelter	Tampa	Time
Last	Tampa	Beauti	Shift	Tri	Night
Shelter	Last	Let	Guess	Time	Make
Make	Read	Storm	Tri	Made	Friend
Night	Outsid	Night	Last	Yet	Alway
Beauti	Check	Tomorrow	Pet	Whole	Sleep
Yet	Ese	See	Sleep	Night	Need
Close	Made	Tri	Outsid	Tomorrow	Want
Help	Tri	Shelter	Time	Mom	World
Outsid	Tomorrow	#Irma	Check	Friend	Fuck
Hit	Time	Like	Strong	Outsid	Wind
Whole	Sleep	Hurrican	Made	Make	Watch
Tri	Night	Last	Cuba	Alway	Boy
#Irmahurrican	Make	Make	Help	Sleep	Nigga
Time	Yet	Still	Saturday	Open	See
Move	Food	Watch	Night	Could	Pleas
Ago	Footbal	Place	Dawg	Hit	Wait
Tomorrow	Might	Need	Yet	Great	Beach
Wait	Lake	Tampa	Eye	Want	Tonight
#Hurricanirma	Spend	Person	School	Need	#Irma

**Table 9:** Related words 06:00 UTC–12:00 UTC.

06:00	07:00	08:00	09:00	10:00	11:00	12:00
Tri	Sleep	Last	Ese	Ese	Tampa	Tampa
Time	Offici	Outsid	Outsid	Tri	Yet	Time
Night	Need	Sleep	Moder	Help	Time	Check
Close	e	Heavi	Valkaria	Close	Ese	Ese
Outsid	Want	e	Sleep	Outsid	Eye	Tri
Sleep	Hit	#Key	Nation	Eye	Friend	Might
Alway	#Key	Wind	e	Moder	First	Night
Need	Wind	Tropic	Need	Sleep	Night	First
Want	Tropic	Good	Wind	Heavi	Last	Close
Well	See	Beach	Fuck	Wellington	Close	Coffe
e	Much	#Irma	#sfltraffic	Wind	#Traffic	Friend
Wind	#Irma	Florida	Pleas	Fuck	Strong	Help
Fuck	Beach	Storm	Storm	Tropic	Outsid	Last
Watch	Florida	#mfl	Beach	Good	Make	Follow
Wait	Storm	Aso	Peopl	See	Well	Outsid
Good	Know	Lauderdal	#Irma	Pleas	Want	Make
Beach	#mfl	Power	Florida	Storm	Phone	Sleep
Storm	Power	Mesonet	f	Flood	Sleep	Strong
Live	Call	Rain	Rain	Beach	Hit	#Irmageddon
Florida	Aso	Safe	Mesonet	Rain	Florida	Open

**Table 10:** Related Words 13:00 UTC–18:00 UTC.

13:00	14:00	15:00	16:00	17:00	18:00
Shelter	Shelter	Shelter	Shelter	#Hurricaneirma	Hit
First	Whole	Tampa	Want	Outsid	Safe
Wait	Tampa	Beauti	Time	Food	Outsid
Beauti	Yet	See	Good	Get	Open
Tri	Check	#Hurricaneirma	Tampa	Safe	Updat
Make	Open	Prep	Get	Watch	Hurrican
Could	Hit	Food	Guess	Time	Make
Made	Get	Come	Hurrican	Peopl	Prayer
See	Friend	Watch	Last	Know	First
Eye	Read	Yet	Check	See	Get
#Hurricaneirma	World	Time	Hit	Love	Wait
World	Safe	Sleep	Peopl	Power	Everyon
Night	Good	Ride	Come	Gonna	Check
Peopl	Time	First	Eye	#Irma	See
Close	Come	Get	Friend	Still	Home
Outsid	Make	Check	Food	Hurrican	Power
Help	First	Go	Day	#nfl	#Hurricaneirma
Landfal	Beauti	Know	See	Make	Okay
Come	Wait	Open	Make	Home	Watch
Pleas	Home	Tri	Way	Want	Yet

**Table 11:** Related Words 19:00 UTC–00:00 UTC.

19:00	20:00	21:00	22:00	23:00	00:00
Tampa	#Hurricaneirma	Shelter	#Hurricaneirma	#Hurricaneirma	Power
Eye	Go	Hurrican	Day	Get	Back
Bay	#Irma	#Irma	Watch	Still	Get
First	Come	Still	Live	Go	Come
Time	Watch	Come	Time	Updat	Even
Wait	#Napl	Updat	Updat	Wait	Got
Hit	Wait	Time	Get	Power	Updat
Outsid	Live	Whole	Make	Last	#Irma
#Hurricaneirma2017	Pass	#Hurricaneirma	Shelter	#Irma	Time
Make	Hit	Make	Go	First	Outsid
Food	Right	Watch	Wait	Yet	Still
Us	Friend	Wait	See	Right	Go
Shelter	Day	Stay	Still	Time	Storm
Get	Shelter	See	#Irma	Tampa	Much
Last	Get	Safe	Hit	Us	Hurrican
Point	Safe	Rain	Need	Everyon	Light
Safe	Beauti	Home	Rain	Home	Let
Open	Look	Tampa	Safe	Made	Friend
Alway	Everyon	Get	Open	Hour	Watch
Video	Make	Pleas	Pleas	Back	Start

#### Graphs of word communities

**Figure 4:**
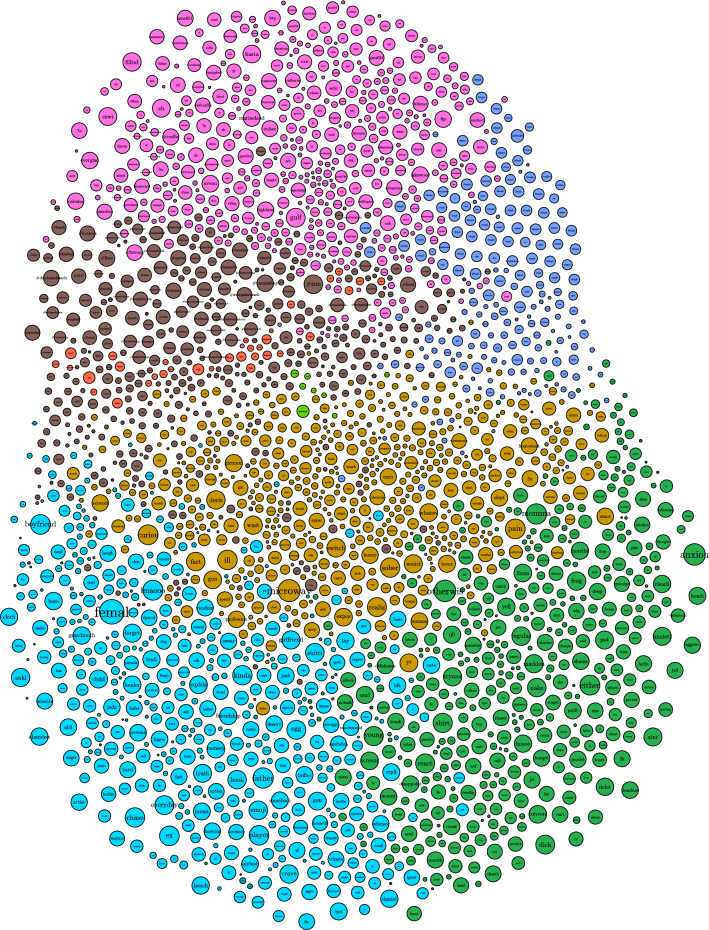
Graph of topic communities.

We explored how different words are connected to each other using word community graphs Each word in Fig. 4, is connected to other terms based on cosine similarity. The edges in this graph represent values for cosine similarity greater than $\cos (45)$ or $\approx 0.7071$. This value was chosen as a lower bound on vector representation of similarity, as included values would be closer to coincident than orthogonal. The nodes are subjected to a gravity algorithm to encourage similar terms to cluster, and dissimilar terms to repel each other. The edges in this graph represent the cosine similarity between the vectors that represent the word embeddings of the words in the nodes. Each node’s relative size is proportional to the related token’s PageRank score.

**Figure 5:**
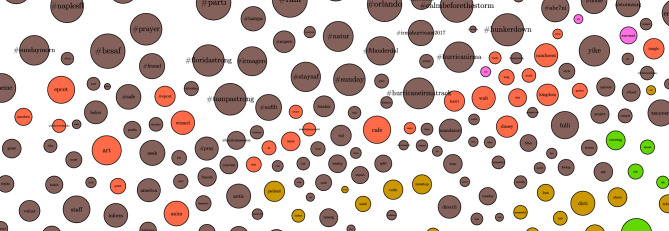
Graph of topic communities: attractions.

**Figure 6:**
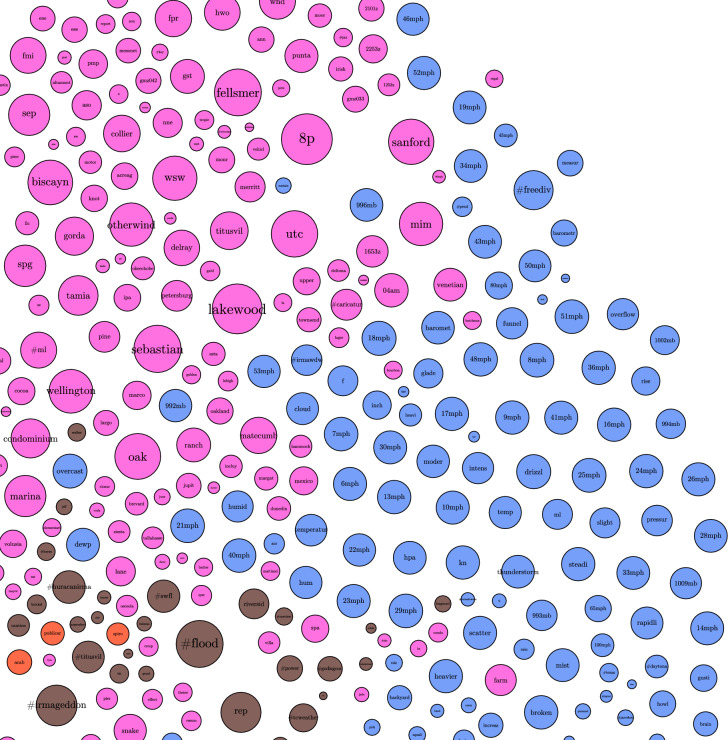
Graph of topic communities: weather.

In the graph, sections separated by color are designated based upon Louvain Modularity. The communities that formed depict topics, with some highlights in the figures below. For example, in Fig. 5, there is the topic of famous Florida attractions as represented by the words: Magic Kingdom, Walt Disney World, Harry Potter’s Wizarding World, and Hollywood Studios. Similarly, in Fig. 6, there appear to be weather related words associated with windspeed (41 mph, 80 mph), pressure (994 mb, 1002 mb, baromet[ric], pressur[e]), weather phenomena (thunderstorm, gust, funnel, squall, drizzl[e], rain, mist, humid[ity]), measurements of compass direction (e, ese, sw, ene, nne), and terms of scale (light, heavi[est], moder[ate], intens[e]).

## Conclusions

This paper tackles the challenge of using social media content, especially Twitter, for emergency response use during disasters. We explore mechanisms for identifying and ranking the most relevant tweets related to a specific search term. We use hurricane Irma as a use case and demonstrate methods for identifying relevant tweets by optimizing different parameters.

In particular, we demonstrate how to train neural networks using either the Continuous Bag-of-Words or the Skip-Gram model. Preprocessing steps such as removing stop words and subsampling frequent words in the tweet corpus helped reduce the number of relevant tokens to enhance retrieval of appropriate tweets.

Comparisons of different scalar formulas were conducted across several tuning parameters. We found that Dot Product with a word window size of 8 resulted in the maximum AU_ROC. We saw that the appropriate minimum word frequency varied depending on the scalar comparison formula. The optimum value for minimum word frequency for Dot Product was found to be 3 whereas the optimal value for all other formulas was 8. This indicates that the performance of the model is tied to the scalar comparison used and its optimal setting. The default setting of 100 dimensions proved to be adequate for the hidden layer dimensionality setting. Negative sampling showed substantial improvements across all scalar comparison formulas between 0 to 1 indicating a minimal number of negative context words in the training has an overall positive effect on the accuracy of the neural network. The methods proposed here are generalizable to a variety of scenarios and applications. They can be used for a variety of social media platforms and can function as a way for identifying the most relevant material for any search term during natural disasters. These approaches once incorporated into digital apps can be useful for first responders to identify events in real time and devise rescue strategies.

## Data Availability

The datasets used and/or analysed during the current study available from the corresponding author on reasonable request. The code used for data analysis can be accessed publicly through our (https://github.com/brownworth/TwitterNLP) repository.
